# A dataset of road-killed vertebrates collected via citizen science from 2014–2020

**DOI:** 10.1038/s41597-022-01599-6

**Published:** 2022-08-17

**Authors:** Florian Heigl, Norbert Teufelbauer, Stefan Resch, Silke Schweiger, Susanne Stückler, Daniel Dörler

**Affiliations:** 1 Institute of Zoology, University of Natural Resources and Life Sciences Vienna, Gregor-Mendel-Straße 33, 1180 Vienna, Austria; 2 BirdLife Österreich, Museumsplatz 1/10/7-8, 1070 Wien, Austria; 3 apodemus–Privates Institut für Wildtierbiologie OG, Marktstrasse 51, 8967 Haus im Ennstal, Austria; 4 First Zoological Department, Herpetological Collection, Natural History Museum Vienna, Burgring 7, 1010 Vienna, Austria

**Keywords:** Biodiversity, Research data

## Abstract

Data on road-killed animals is essential for assessing the impact of roads on biodiversity. In most European countries data on road-killed huntable wildlife exists, but data on other vertebrate species (e.g. amphibians, reptiles, small mammals) is scarce. Therefore, we conducted a citizen science project on road-killed vertebrates as a useful supplement to data on huntable wildlife collected by public authorities. The dataset contains 15198 reports with 17163 individual road-killed vertebrates collected by 912 participants. The reports were made in 44 countries, but the majority of data was reported in Austria. We implemented a data validation routine which led to three quality levels. Reports in quality level 1 are published via GBIF, reports in quality level 2 via Zenodo and reports in quality level 3 were deleted. The dataset is relevant for the scientific community studying impacts of roads on fauna as well as for those who are responsible for road planning and implementing mitigation measures.

## Abstract (summary)

**Table:** 

Measurement(s)	vertebrate roadkills
Technology Type(s)	online forms and apps for android and iOS
Sample Characteristic - Organism	Vertebrate

## Background & Summary

Biodiversity is declining globally. This process is seen as a global crisis which, in combination with climate change, is expected to lead to dramatic changes in our ecosystems^1^. The global road network is increasing, leading to a vast environmental impact on biodiversity^2,3^, ranging from indirect impacts such as light pollution or air pollution to more direct impacts such as habitat fragmentation and habitat loss^4^. Roadkill is one of the most direct negative impacts of road traffic on vertebrate species^5^. However, data on road-killed animals on a large scale is scarce, especially regarding non-huntable wildlife. In Austria, for example, only data on road-killed huntable wildlife is gathered on a regular basis by hunters and summarised by the federal institute on statistics, Statistics Austria^6^. Besides nature conservation, data on road-killed animals are also important for traffic safety. In general, vertebrates on roads pose a significant risk for drivers. Large vertebrates (e.g. Red deer, Wild boar) can cause accidents by direct collisions and smaller vertebrates can lead to evasive manoeuvres by drivers^7–10^.

To supplement official data on huntable wildlife killed on roads with data on non-huntable wildlife, we launched the citizen science project ‘Roadkill’ in 2014 (https://roadkill.at/en). The project allows registered users to report so-called presence-only data on road-killed animals during their daily routine (e.g. commuting, cycling, hiking) with an online-form, or apps for iOS and Android devices. The reports are visualised immediately in the interactive map on the project website and in the apps, which provide feedback to the citizen scientists and contributes to raise awareness for the problem of road-killed animals, through understanding that their reported animal is not an isolated incident, but part of a larger picture.

Our dataset contains information on various vertebrate species. It thus can provide important information for planning mitigation measures, as ecological factors influencing the number of roadkills depend on the species studied^11–13^. So far, four peer-reviewed articles were published based on the dataset. In 2016 we compared reports on road-killed European hares from our citizen science project with data collected by hunters. Our results indicated that hunters tend to report data mainly from their hunting areas, whereas citizens report data during their daily routine on the way to/from work. It was concluded that a citizen science approach is an important source for roadkill data when used in addition to official data with the aim of obtaining a more complete overview of road-kill events^14^. In 2017 hotspot analyses of amphibians and reptiles in Eastern Austria revealed significant clustering of road-killed amphibians and reptiles, which is important information for authorities aiming to mitigate roadkills^15^. In 2020, during the COVID-19 lockdowns, we investigated if the observed decrease in roadkill reports was grounded in less animals being killed by traffic, or in citizen scientists staying at home and thus reporting less road-killed animals. The survey results suggest that a majority of the respondents have reported less roadkills during the lockdown, regardless if they changed their travelling routine or not. The survey results combined with the general decrease in road traffic, suggested that fewer animals were killed during the lockdown^16^. To predict amphibian migration events in spring, we compared the migration of common toads (*Bufo bufo*) and common frogs (*Rana temporaria*) with the phenology of five tree, one shrub, and one herb species. The results showed a close association between common frog migration and the phenological phases of European larch (*Larix decidua*, Mill.), goat willow (*Salix caprea*, L.) and apricot (*Prunus armeniaca*, L.). Thus, plant phenology seems to be useful to determine the onset of temporary protection measures for certain amphibian species to prevent roadkills^17^.

Following Project Splatter (United Kingdom) and the Taiwan Roadkill Observation Network^18,19^, we too published the dataset via GBIF and Zenodo. To the best of our knowledge, our dataset is the first dedicated exclusively to road-killed animals in continental Europe which is openly available.

The dataset is useful for global impact studies of road-killed animals on biodiversity as well as for local public authorities, which investigate potentially dangerous road segments. Additionally, the dataset in GBIF is used for studies outside the field of road ecology and thus this project also contributes to biodiversity research^20^.

## Methods

### Data collection

The dataset presented was collected via a citizen science approach. We chose citizen science to collect data on road-killed animals, since it is the most effective method to collect data on a large scale, and also because it’s widely accepted in the scientific community^21–26^. Although no generally accepted international definition of citizen science exists^27–29^, we define citizen science as the active involvement of people in scientific projects without project-specific professional and scientific background^30,31^. In our project citizens were mainly involved in data collection. Citizen scientists collected data on road-killed animals during their daily routine using smartphone apps for Android and iOS devices, or the online form on the project’s website.

**Fig. 1:**
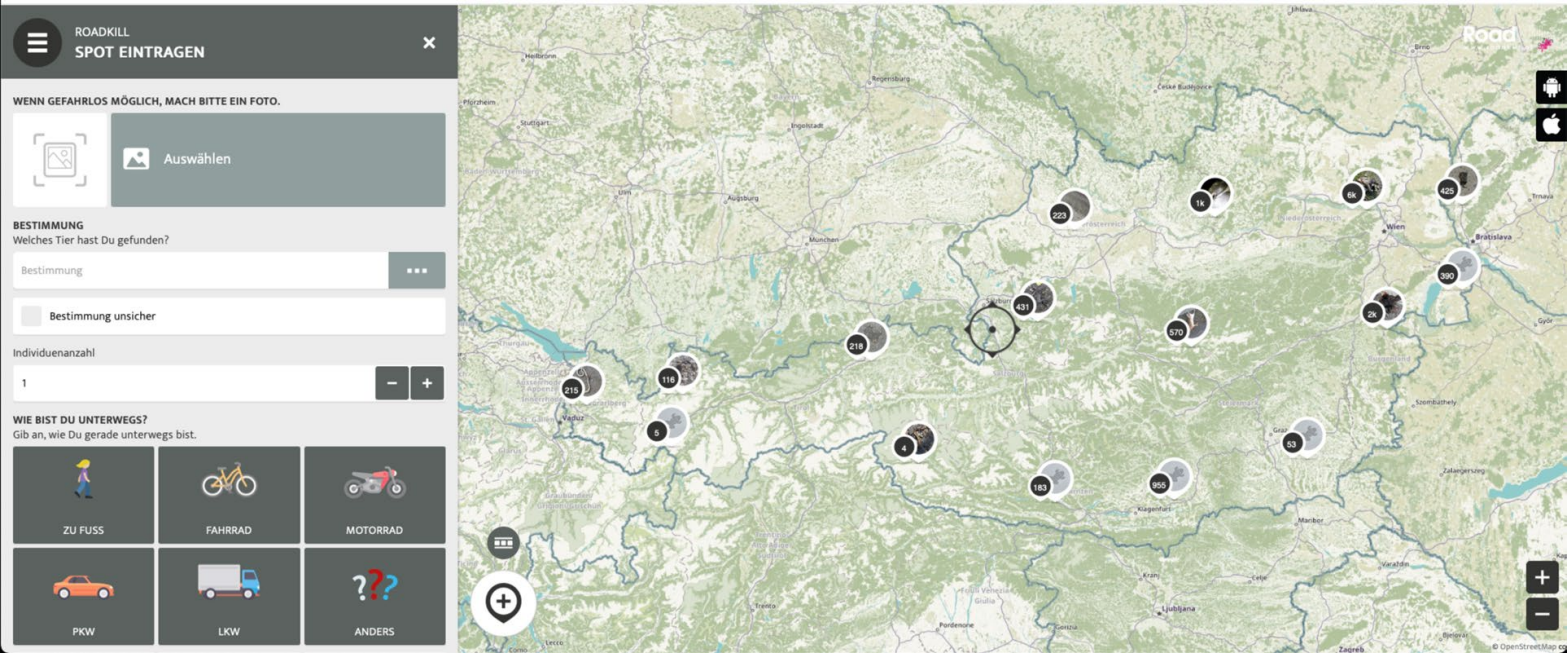
Screenshot of the online data-submission form. On the left side you can see the form where you can upload photos, determine the roadkill and make other specifications. On the right side you see the Open Streep Map (openstreetmap.org), with the crosshairs in the middle, with which you can indicate the position of the report. You can zoom in and out on the map to indicate the exact position. In the map you can also see all the reports that have been entered so far in order to avoid duplicate reports.

We launched the citizen science project Roadkill in 2014 to investigate which vertebrate species are killed on which locations on Austrian roads^32^. The technical system and used software of data collection changed over time, but the main fields of enquiry (e.g. picture, organism, coordinates, date, number of individuals) in the submission form stayed the same (Fig. 1 and Supplementary Table 1).

A detailed description of the data collection systems can be found in^14,15,32^. Since 2015 we use smartphone apps and online forms developed by Spotteron GmbH | Citizen Science platform (https://www.spotteron.net/) in German and English for data collection. Interested people are able to join the project by registering on the project website (https://roadkill.at/en/) or directly in the SPOTTERON Roadkill app for Android or iOS systems (https://roadkill.at/en/home-en). After successful registration, participants can report data via the submission form on the website or in the app. Data submission without registration or login is not possible. Each report entered in the database appears immediately on an interactive map on the project’s website.

## Data Records

**Fig. 2:**
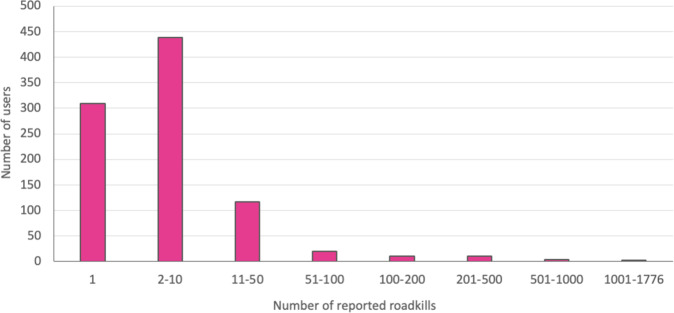
Number of reported roadkills per user.

The presented dataset has been released via GBIF (https://www.gbif.org)^33^ and Zenodo (https://zenodo.org/)^34^ and it contains 15198 reports with 17163 individual road-killed animals collected by 912 participants from February 2014 - December 2020. With one report several individuals can be reported at the same time, therefore the number of reports is less than individual roadkills. When participants in Project Roadkill are grouped by number of reported roadkills, a typical picture of crowdsourcing projects can be seen^35^ (Fig. 2). The majority of citizen scientists report single roadkills and few citizen scientists collect large amounts of data.

**Fig. 3:**
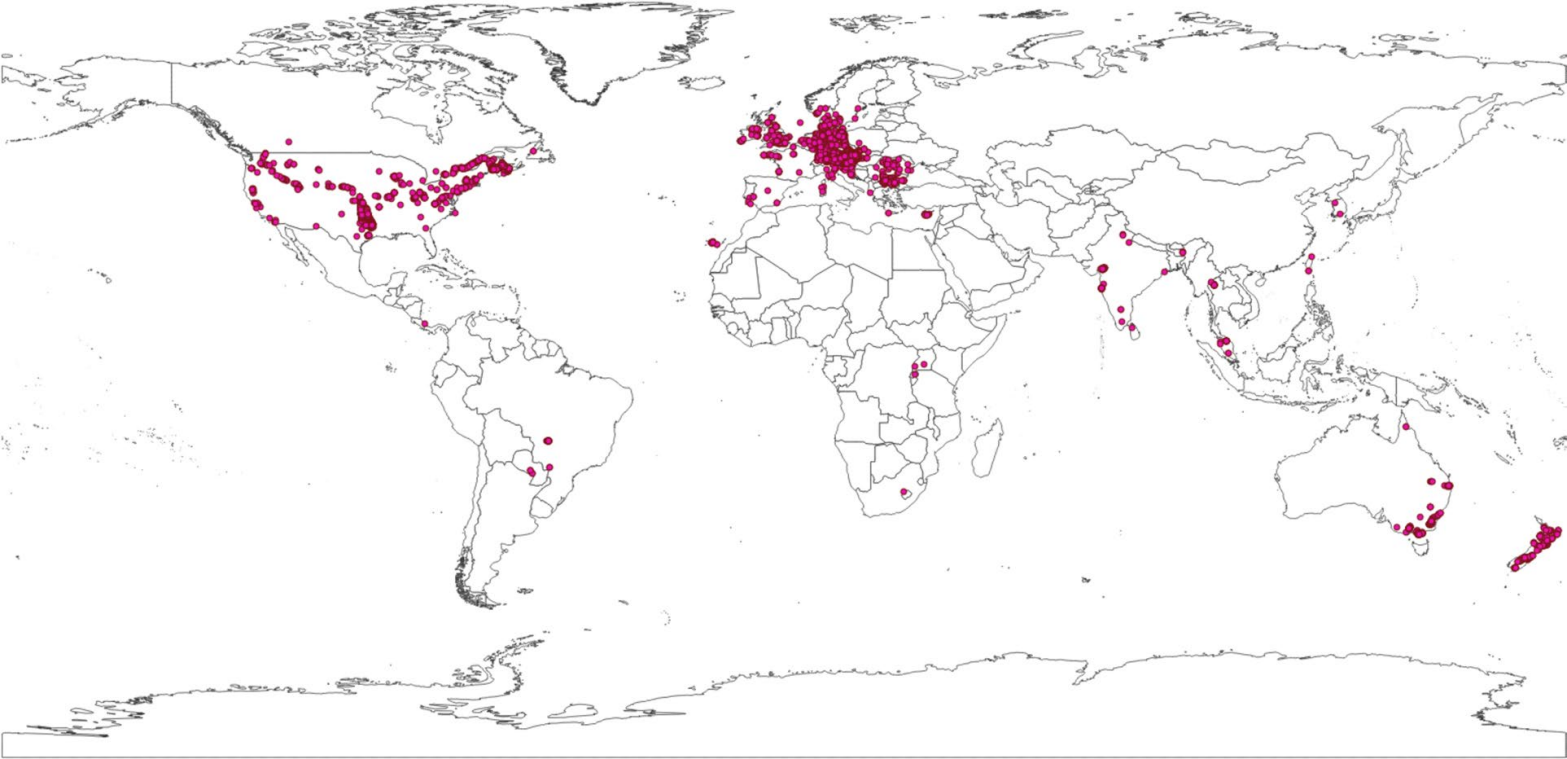
Global map showing the distribution of reported road-kills. The underlying world map is provided under a CC0 license by Natural Earth (naturalearthdata.com).

Data collection from 2014–2020 resulted in data on road-killed animals from 44 countries. However, the focus of data collection was in Austria (Fig. 3).

### Protected species

To provide an overview of how many of the reported individuals are endangered species, we grouped all data - where the animal could be identified to species level - according to the IUCN Red List classification^36^. 466 individuals belong to species which are listed between the status “Near Threatened (NT)” and “Critically Endangered (CR)”, 8629 individuals belong to species listed in the status “Least Concern” (Table 1). Overall 4048 individuals belong to species where the status is stated as decreasing, meaning that the numbers of individuals of this species are globally declining.

**Table 1:** Data of species listed in the IUCN red list of threatened species in the categories Least concern (LC), Near threatened (NT), Vulnerable (VU), Endangered (EN), Critically endangered (CR).

Red List Category	Trend											
	increasing		stable		decreasing		unknown		no information		Total	
	Species	Indiv.	Species	Indiv.	Species	Indiv.	Species	Indiv.	Species	Indiv.	Species	Indiv.
LC	48	1448	80	3223	66	3589	11	92	1	277	206	8629
NT	1	2	—	—	9	184	2	5	—	—	12	191
VU	—	—	—	—	7	10	—	—	—	—	7	10
EN	—	—	—	—	2	189	—	—	—	—	2	189
CR	—	—	—	—	1	76	—	—	—	—	1	76
No information	—	—	—	—	—	—	—	—	—	—	17	695
Total	49	1450	80	3223	85	4048	13	97	1	277	245	9790

#### Dataset availability

The dataset containing data with quality level 1 can be accessed and downloaded via GBIF^33^. The dataset containing data with quality level 2 can be accessed and downloaded via Zenodo^34^.

Preferred Identifier: DOI

## Technical Validation

### Routine data validation

As soon as a citizen scientist reports a roadkill, it is visible on the project website. The reports can be filtered by species, a time series and a so-called heatmap can be created where reports are clustered spatially. These features are used by citizen scientists to check the data and comment on reports if they find conspicuities. These conspicuities are either corrected by the citizen scientist who reported the roadkill or is taken care of by the project team. Every second day, the data entered into the Roadkill project is validated to correct false or conspicuous entries via the backend of the website. If the report cannot be corrected, it is deleted. Correction of data was done (i) by the project team itself if errors were obvious (e.g., animal in submitted image does not match species identification listed) or (ii) by the participants themselves after being advised by the project team that a correction was needed (e.g., roadkill is not on a road).

**Fig. 4:**
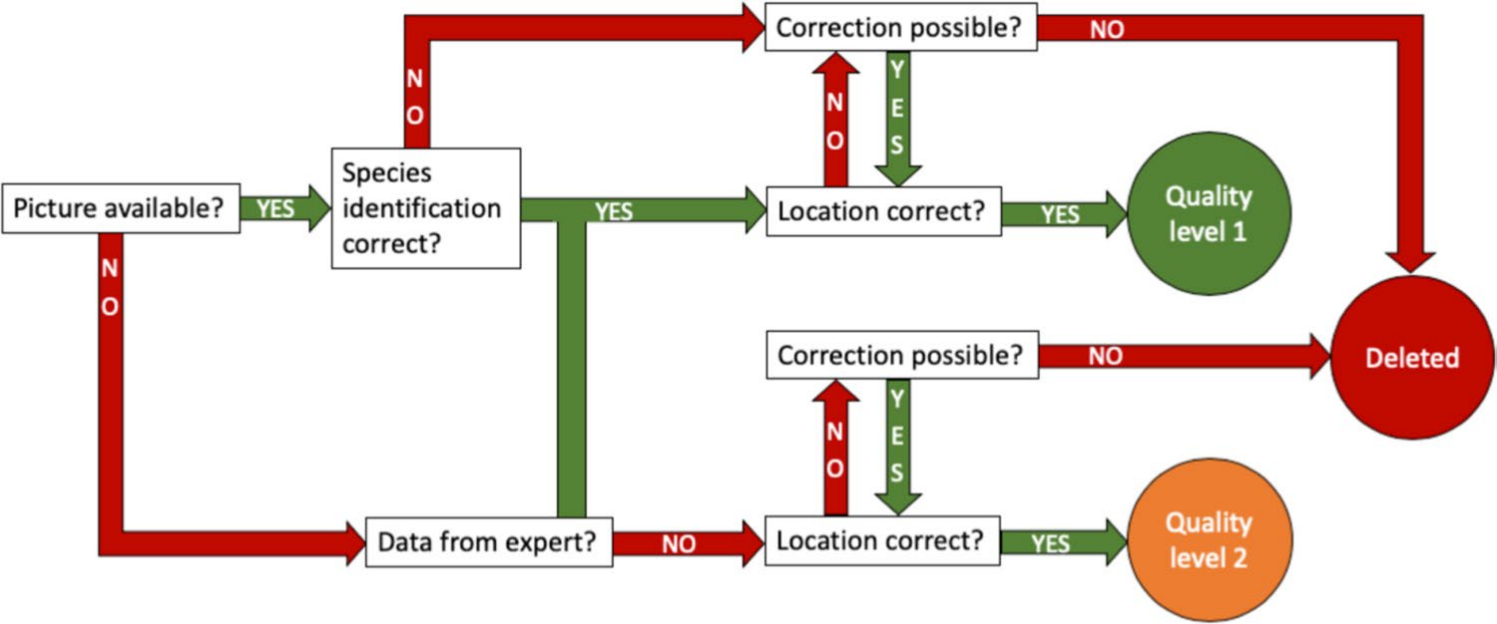
Scheme for categorizing the data submitted to the project Roadkill in three different quality levels.

To ensure the quality of the data, we used a stepwise selection process that allowed us to classify the submitted data into three quality levels at the end of this process (Fig. 4):

- Quality level 1: Data sets with correct animal identification (either the dataset was reported by an expert or animal in submitted image matches species identification listed) and inconspicuous data.
- Quality level 2: Data sets with inconspicuous data but no possible validation of the animal.
- Deleted: records with conspicuous data and no possible validation of the animal.

This routine has the advantages that it can be carried out quickly in practice, is comprehensible and can therefore be used by several people in the team. Difficulties have arisen with the photos, as the quality of the photos does not always allow an exact identification of the animals (photo is blurred, animal is too far away, animal is too destroyed). If the photo does not allow validation, the report was treated the same as a report without a photo. Reports without photos were only counted as quality level 1 if they came from experts known to us with expertise in the reported animal group. Experts can be, for example, ecologists/zoologists or members of nature conservation organisations.

Of the total 15819 reports, 8347 reports (52.77%) were quality level 1 and published on GBIF, 6851 reports (43.31%) were quality level 2 and published on Zenodo and 621 reports (3.92%) were deleted. The main reasons for deleting a report were (1) the report was positioned offroad in our map, (2) the report was no roadkill, but a species killed by another reason (e.g. a bird that flew against a window pane next to a parking lot) or (3) we did not receive any feedback on a question about an inconspicuous report. Wherever possible, we corrected reports with obvious implausibilities (2663 reports, 17.52%). Most of the corrections were needed due to errors in locations (1550 reports, 10.2%), meaning the location of the report on our map was slightly beside the road. We corrected the location only in cases where no misinterpretation of the location was possible, e.g. a report between two roads was deleted, but a report a few metres next to a road was moved to the road. Some animals stray from the road after being hit, but we ask our participants to enter the report where the animal was hit on the road. For example, if the animal is found in a field next to a road, the report will still be on the road. If participants submitted a report with an uncertain species identification and we could verify the species identification, we changed the status of the report to “certain” (843 reports, 5.55%). We corrected species identification in 626 reports (4.12%). In 359 reports (2.36%) we corrected a combination of the above-mentioned aspects.

### Additional manipulation of the dataset

The drop-down menu in the project’s online form and app contains Austrian vertebrate species only. If citizen scientists find an animal not listed, they have to select e.g. “other mammal” and type in the species name manually. Therefore, we changed reports containing “other” vertebrate species to a more detailed identification if possible. We changed all reports from New Zealand identified as “possum” to Common brushtail possum (*Trichosurus vulpecula*) and all reports from North America identified as “possum” to Virginia opossum (*Didelphis virginiana*). Since all “porcupines” were reported from North America, we changed these entries to North American porcupine (*Erethizon dorsatum*).

## Supplementary information

Supplements

## Data Availability

For data collection we used the commercial software SPOTTERON by the company SPOTTERON GmbH (https://www.spotteron.com). The code for this software is not open source.
