# Supplementary material for: A dataset of road-killed vertebrates collected via citizen science from 2014–2020
Source: Sci Data. 2022 Aug 17;9:504. doi: 10.1038/s41597-022-01599-6 (PMC9383656; doi:10.1038/s41597-022-01599-6)
Supplement: Supplementary file 1 — Supplements [file 41597_2022_1599_MOESM1_ESM.pdf]

Supplementary Table 1: Table of enquiry fields

Supplementary Table 2: List of species from the dropdown menu in the project Roadkill application and online form.

Supplementary Table 1: Table of enquiry fields

**Field name**	**Mandatory**	**Description of field**	**Answering mode**	**Answering options**
Location	yes	Participants give the exact location of the road-killed animal	GPS coordinates	• automatically by using the GPS-location of the smartphone<br>• manually by setting a point on a map
Picture	no	Participants make or upload a picture of the road-killed animal (if possible)	n/a	n/a
Identification	yes	Participants identify the animal as accurate as possible (from class to species)	single choice	Species List (see Supplementary Table 2)
Identification certainty	no	Participants can indicate if they are unsure that the identification is correct	single choice	Identification uncertain
Number of individuals	yes	Participants give the number of animals they found at a certain location	single choice	1-
Modes of transportation	yes	Participants specify which means of transportation they were using when finding the roadkill	single choice	• by foot<br>• by bike<br>• by motorbike<br>• by car<br>• by bus<br>• others
Frequency of travels	yes	Participants specify how often they travel on this road	single choice	• one-time travel<br>• rarely (once a month)<br>• 1-2 times per

				week<br>• 3-5 times per week<br>• daily
Description	no	Participants can give additional information they feel is important	free text	

Supplementary Table 2: List of species from the dropdown menu in the project Roadkill application and online form.

**Identification level 1**	**Identification level 2**	**Identification level 3**
Mammal	Beaver	
	Squirrel	
	Moose	
	European hare	
	Bat	
	Fox	
	Chamois	
	Deer	
	Dog	
	Hedgehog	
	Cat	
	Small mammal	Murinae
		Arvicolinae
		Shrew
		Dormouse

		Dipodidae
		Hamster
		Mole
	Mustelidae	Stoat
		Least weasel
		Polecat
		Pine marten
		Beech marten
		Badger
		Otter
	Mouflon	
	Marmot	
	Roe deer	
	Ibex	
	Raccoon	
	European rabbit	
	Wild boar	
	Spermophilus	
	Other mammal	
Amphibians	Frogs and Toads	Fire-bellied Toad
		Yellow-bellied Toad
		Common Spadefoot Toad

		Common Toad
		Natterjack Toad
		European Green Toad
		European Treefrog
		Moor Frog
		Agile Frog
		Common Frog
		Green Frogs
	Newts	Alpine Newt
		Triturus
		Smooth Newt
	Salamanders	Alpine Salamander
		Fire Salamander
	Other amphibians	
Reptile	Slow Worm	
	True Lizards	
	Turtles	
	Snakes	Smooth Snake
		Aesculapian Snake
		Grass Snake
		Dice Snake
		Common European Adder

		European Nose-Horned Viper
	Other reptiles	
Bird	Size of sparrow	
	Size of blackbird	
	Size of dove	
	Bigger as dove	
